# Inflammatory Dietary Potential Is Associated with Vitamin Depletion and Gut Microbial Dysbiosis in Early Pregnancy

**DOI:** 10.3390/nu16070935

**Published:** 2024-03-24

**Authors:** Suzanne A. Alvernaz, Elizabeth S. Wenzel, Unnathi Nagelli, Lacey B. Pezley, Bazil LaBomascus, Jack A. Gilbert, Pauline M. Maki, Lisa Tussing-Humphreys, Beatriz Peñalver Bernabé

**Affiliations:** 1 Department of Biomedical Engineering, University of Illinois, Chicago, IL 60607, USA; salver5@uic.edu (S.A.A.); usvnagelli@gmail.com (U.N.); 2 Department of Psychology, University of Illinois, Chicago, IL 60607, USA; ewenze4@uic.edu (E.S.W.); pmaki1@uic.edu (P.M.M.); 3 Department of Kinesiology and Nutrition, University of Illinois, Chicago, IL 60612, USA; lwissl2@uic.edu (L.B.P.); bbrain2@uic.edu (B.L.); tussing@uic.edu (L.T.-H.); 4 Department of Pediatrics, University of California, San Diego, CA 92093, USA; jagilbert@health.ucsd.edu; 5 Scripps Oceanographic Institute, University of California, San Diego, CA 92037, USA; 6 Department of Psychiatry, University of Illinois, Chicago, IL 60612, USA; 7 Department of Obstetrics and Gynecology, University of Illinois, Chicago, IL 60612, USA; 8 Center for Bioinformatics and Quantitative Biology, University of Illinois, Chicago, IL 60612, USA

**Keywords:** diet, inflammation, pregnancy, gut microbiota, dietary inflammatory index, galactose, food frequency questionnaire

## Abstract

Pregnancy alters many physiological systems, including the maternal gut microbiota. Diet is a key regulator of this system and can alter the host immune system to promote inflammation. Multiple perinatal disorders have been associated with inflammation, maternal metabolic alterations, and gut microbial dysbiosis, including gestational diabetes mellitus, pre-eclampsia, preterm birth, and mood disorders. However, the effects of high-inflammatory diets on the gut microbiota during pregnancy have yet to be fully explored. We aimed to address this gap using a system-based approach to characterize associations among dietary inflammatory potential, a measure of diet quality, and the gut microbiome during pregnancy. Forty-seven pregnant persons were recruited prior to 16 weeks of gestation. Participants completed a food frequency questionnaire (FFQ) and provided fecal samples. Dietary inflammatory potential was assessed using the Dietary Inflammatory Index (DII) from the FFQ data. Fecal samples were analyzed using 16S rRNA amplicon sequencing. Differential taxon abundances with respect to the DII score were identified, and the microbial metabolic potential was predicted using PICRUSt2. Inflammatory diets were associated with decreased vitamin and mineral intake and a dysbiotic gut microbiota structure and predicted metabolism. Gut microbial compositional differences revealed a decrease in short-chain fatty acid producers such as *Faecalibacterium*, and an increase in predicted vitamin B12 synthesis, methylglyoxal detoxification, galactose metabolism, and multidrug efflux systems in pregnant individuals with increased DII scores. Dietary inflammatory potential was associated with a reduction in the consumption of vitamins and minerals and predicted gut microbiota metabolic dysregulation.

## 1. Introduction

In pregnancy, an under- or oversupply of nutrients can have deleterious impacts on both maternal and fetal health. For instance, a lack of adequate folic acid intake during pregnancy is one of the leading causes of neural tube defects during fetal development [1]. Similarly, iron utilization increases during the course of pregnancy, and an inadequate supply is associated with poor fetal outcomes, including intrauterine growth restriction and low birth weight [2]. Conversely, an oversupply of dietary nutrients, including carbohydrates and saturated fats, common in Western diets, are associated with chronic inflammation and can lead to obstetric complications, from gestational diabetes mellitus (GDM) [3,4] to preterm birth [5]. This is especially important for minoritized women of color who may have a poor nutritional intake due to structural inequalities [6,7,8] and experience a higher burden of adverse pregnancy outcomes [9]. Thus, understanding the pro-inflammatory nature of diets could serve to reduce negative obstetrics and delivery outcomes [10]. 

Diet is a major regulator of the gut microbiota [11,12]. The gut microbiota encompasses the bacteria, fungi, viruses, and protists living inside the human gastrointestinal tract. It is estimated that the combined genomes of all gut bacteria comprise >5 million genes [13], with the potential to metabolize a vast number of different substrates. An Over- or undersupply of dietary nutrients (such as fats or fiber) can provide competitive advantages or disadvantages for different gut microbial species based on their individual metabolic capabilities [14,15]. The dynamic nature of pregnancy alters almost every system in the body, including the maternal gut microbiota [16] and immune system [17], which adapts in time with a tightly regulated clock to maintain the immune protection of the mother while simultaneously avoiding the autoimmune rejection of the growing fetus [17,18]. The structure of the gut microbiota changes as the pregnancy progresses [19,20,21]. In fact, the transplantation of gut microbiota from pregnant individuals into germ-free animals renders common pregnancy phenotypes of obesity, insulin resistance [19,22], and adaptations in immunity [23]. A poor diet quality leading to a pro-inflammatory state can alter the normal dynamic changes in the gut microbiota [14] and immune system during pregnancy [24], increasing the risk of common perinatal complications, including GDM [25], iron deficiency [26], and mood disorders [27]. It is thus essential to understand how maternal diet quality during pregnancy impacts the gut microbiota. 

The Dietary Inflammatory Index (DII) is a literature-derived population-based index used to quantify the inflammatory potential of diets among diverse populations [28]. The index was developed by leveraging global dietary studies to assign inflammatory effect scores (*Ss*) to common dietary nutrients based on their ability to increase pro- or decrease anti-inflammatory biomarkers, such as cytokines IL-1β, IL-6, IL-4, and IL-10 [28,29,30]. Previous studies have shown that the DII is positively associated with inflammatory markers during pregnancy [31], increased rates of cesarean delivery in obese mothers [32], and decreased fetal growth [33]. Furthermore, the DII has also been negatively linked with microorganisms that produce short-chain fatty acids (SCFAs), which are beneficial anti-inflammatory metabolites [34,35,36,37]. Thus, the normal gut microbial compositional changes occurring during the gestational period may be negatively altered by a poor diet quality, which could be assessed using the DII score, and may mediate obstetric complications. 

Dysbiosis refers to the imbalance in the gut microbiota, where the equilibrium between beneficial and harmful microorganisms is disrupted by factors such as poor diet, antibiotics, or illness [25]. Microbial dysregulation, closely related to dysbiosis, describes when the regulation of these microbial populations is disturbed, leading to health issues [27] that can manifest as an impaired immune response or altered metabolic processes [21]. Both dysbiosis and microbial dysregulation are crucial concepts in understanding conditions like obesity and gestational diabetes, where the gut microbiota plays a significant role in disease progression or mitigation [21]. Understanding how diet regulates the gut microbiota during pregnancy could potentially lead to avenues of early interventions to reduce the risk of pregnancy comorbidities associated with systemic inflammation. Here, we aimed to assess the relationship between dietary inflammatory potential and the maternal gut microbiota during the first trimester of pregnancy in a cohort mostly composed of minoritized women of color living in a large diverse urban community in the United States. We hypothesized that there would be an increase in bacterial species and predicted microbial metabolic potential associated with inflammatory processes in subjects reporting high DII scores. 

## 2. Materials and Methods

### 2.1. Participant Recruitment 

This work is a secondary data analysis of a longitudinal cohort study (MoMent) in which participants were recruited from the outpatient obstetric clinics at a public university hospital, the University of Illinois Chicago (Chicago, IL, USA), from 2017 to 2020 [38]. This study was approved by the University of Illinois Chicago Institutional Review Board (IRB #2014-0325, 4 September 2014). Written informed consent was obtained prior to study enrollment and sample collection. To be eligible for the study, participants had to be less than 16 weeks pregnant and speak English. Women were excluded for the following criteria: less than 18 or over 64 years of age, current multi-gestational pregnancy, a prior history of gastrointestinal surgeries, oral antibiotic, antiviral, or antifungal use in the last 6 months, use of medication or supplements to treat any chronic disorder (e.g., diabetes, hypertension, mood disorders), history of substance abuse (excluding marijuana, alcohol, and tobacco; self-report) within the last 6 months, use of in vitro fertilization treatments for current pregnancy, active diagnosis of cancer, or HIV or eating disorders or chronic diarrhea within the last 6 months. For this secondary study, we selected participants who completed a diet food frequency questionnaire before 28 gestational weeks and provided a fecal sample at their first study visit (<16 gestational weeks), rendering a total of 47 subjects. See Supplemental Figure S1 for the participant enrollment flowchart. 

### 2.2. Stool Collection 

Study participants self-collected rectal swabs (*n* = 42), avoiding touching the rectal tissue, or provided stool samples (*n* = 5) for gut microbiota assessment. Stool samples were homogenized and aliquoted in cryogenic vials without additives for unaltered sequencing. Rectal swabs and aliquoted stool samples were stored at −80 °C before being sent for 16S rRNA amplicon sequencing. Biological samples were collected with an average estimated gestational age of 10.9 ± 3 weeks. 

### 2.3. Dietary Assessment 

Participants completed one of two validated FFQs: Vioscreen (*n* = 24) [39] or the Diet History Questionnaire II (DHQII) (*n* = 23) [40] with an average estimated gestational age of 14.7 ± 5.9 weeks. Vioscreen is a web-based platform that can assess up to 90 days of intake using nutrition information from the Nutrition Coordinating Center (NCC) Food and Nutrient Database [39]. The DHQII is a paper-based questionnaire that asks 134 food and 8 supplement questions [40]. This platform was created by the National Cancer Institute. Participants were asked about the previous month of dietary intake. The Vioscreen questionnaire was completed electronically at home by participants, with some receiving calls from research staff to complete the survey. The DHQII was completed in-person with a certified registered dietitian within an average of 4.4 ± 5.5 weeks of microbiome sample collection. The Dietary Inflammatory Index (DII) was calculated using the DII components common of both FFQs, with a total of twenty-seven variables (60% of total DII parameters), which is within the DII’s developer’s suggested limit [28]. Individuals were checked to ensure a daily caloric intake of <500 or >5000 kcal/day). The DII variables included were their daily intake of alcohol (g), vitamin B12 (μg), vitamin B6 (mg), β-carotene (μg), caffeine (g), carbohydrates (g), cholesterol (mg), energy (kcal), total fat (g), fiber (g), folic acid (μg), iron (mg), magnesium (mg), monounsaturated fatty acids (myristoleic acid, MUFA 14:1) (g), niacin (mg), total protein (g), polyunsaturated fatty acids (PUFA) (g), riboflavin (mg), saturated fat (g), selenium (μg), thiamin (mg), trans-saturated fat (g), vitamin A (retinol equivalents), vitamin C (mg), vitamin D (μg), vitamin E (mg), and zinc (mg). Individual DII scores were calculated using Equation (1):

(1) $$\mathrm{DII}\;=\;\sum_{i=1}^{n}\left[\left(\left[\phi \left(\frac{\mu_{x_{i}}-\mu_{y_{i}}}{\sigma_{i}}\right)\right]\times 2-1\right)\times S\right]$$

where n represents the total number of common DII parameters between the VioScreen and DHQII; μxi is the mean daily intake of food parameter i obtained from the FFQ; μ_yi_ is the global mean (average daily intake across global populations) and σ_i_ is the global standard deviation of parameter i, both derived from the reference table; Φ is the cumulative distribution function; and S represents the inflammatory effect score. Scores can range from −8.87 to +7.98 with the latter being the most inflammatory [28]. After calculating DII scores for each participant, individuals were grouped into tertiles. 

### 2.4. Dietary Statistics 

Differences in patient demographics by DII tertile were assessed using Chi-square (qualitative) or ANOVA (quantitative) assessments. Correlations among DII parameters (continuous scale) were identified using Spearman’s correlation using energy-corrected nutritional values. Energy correction was performed by scaling each subjects’ food parameter by their reported daily caloric intake. Differences in nutrient parameters by tertile were assessed using ANOVA, and between Tertile 1 and Tertile 2/3, using the student’s *t*-test. All analyses were completed in R. 

### 2.5. Microbiota Assessment 

Rectal and fecal samples underwent 16S rRNA amplicon sequencing in four different batches at the University of Chicago (Chicago, IL, USA) and at the University of California San Diego (San Diego, CA, USA) together with control samples to account for possible reactant and environmental contaminations. Forward raw FASTQ sequences were processed using the DADA2 pipeline independently using default parameters [41] and passed to the R package phyloseq [42]. After primer removal, reads were truncated to 150 base pairs and denoised using standard parameters, and chimeras were removed. A taxonomical assessment of the trimmed, cleaned reads was performed using the Silva reference database version 132 [43]. Contaminating amplicon sequence variances (ASV) found in blank controls were removed from each batch using the prevalence method in the R *decontam* package [44]. A threshold of 0.5 was used to identify contaminants that were more prevalent in negative controls than in clinical samples. Samples with a library size below 10 reads were excluded from downstream analysis. Subsequently, batch-effects were removed using the R package *ComBat-seq* [45]. The count table and taxonomic assignments for each batch were then merged, keeping all the amplicon sequencing variants (ASVs). ASVs with a relative abundance of less than 1% relative to the sample library size were removed from downstream analysis. After prevalence filtering, taxa counts were normalized using cumulative sum scaling (CSS) [46]. 

### 2.6. Microbiota Diversity Assessment

Alpha diversity was calculated using the Shannon [47] and Simpson indexes [48]. Statically significant differences in mean alpha diversity between DII tertiles were assessed using the Wilcoxon rank-sum test [49]. Beta diversity was determined with the Bray–Curtis index [50] and unweighted, normalized UniFrac distance [51]. Significant differences in beta diversity distances based on the DII scores were assessed using PERMANOVA [52], correcting for participant BMI, gestational weeks (EGA), food frequency questionnaire type (DHQII or Vioscreen), sample type (stool or rectal), and maternal age. 

### 2.7. Microbiota Differential Abundance 

Associations between the DII and CSS-normalized ASVs were identified by fitting a zero-inflated Gaussian model with the R package *metagenomeSeq* [53]. Models were adjusted using the same covariates as before. Multiple comparisons were corrected using the Benjamini–Hochberg method [54]. 

### 2.8. Microbiota Predicted Metabolic Potential

Finally, gut metabolic potential was predicted via PICRUSt 2.0 (Phylogenetic Investigation of Communities by Reconstruction of Unobserved States) [55]. Associations between metabolic pathways, microbial enzymes, and DII scores were assessed with zero-inflated Gaussian models, corrected using the same covariates as above and multiple comparisons were adjusted using the Benjamin–Hochberg method. Gene set enrichment analysis (GSEA) was performed using all microbial enzymes, identified as significant before FDR adjustment using the R package *MicrobiomeProfiler* [56]. Finally, associations among the identified enzymes and each food parameter used in the DII estimation were quantified with zero-inflated Gaussian models, corrected using the same covariates as above, and multiple comparisons were adjusted using the Benjamin–Hochberg method. A total of 27 models were fit with Z-scored energy-corrected food parameters per subject with the outcome and microbial enzymes as predictors. 

## 3. Results

### 3.1. Our Sample Was Composed of Minoritized Women of Color with a Large Percentage Consuming a Vitamin-Depleted Pro-Inflammatory Diet

**Figure 1:**
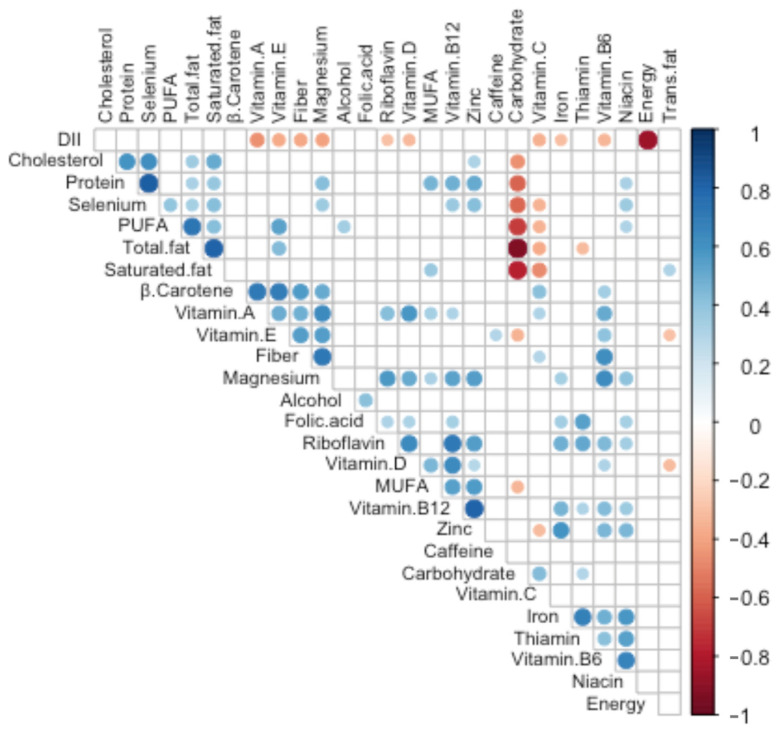
Components and drivers of the DII scores. Spearman correlation (*p* < 0.05) among the 27 parameters used to calculate the DII scores for each subject. Dot size is proportional to the absolute correlation coefficient. See Table 2 for units. PUFA: polyunsaturated fatty acids; MUFA: monounsaturated fatty acids.

A total of 47 participants completed an FFQ and provided a fecal sample. The study cohort was primarily comprised of non-Hispanic Black (44%) and Hispanic (15%) pregnant persons with an average estimated gestational age of 10.9 ± 3 weeks at fecal sample collection, an average maternal age of 29 ± 6 years, and 72% reporting an annual household income below $31,000 per year (Table 1). Notably, most participants reported use of Federal Aid Health Insurance (74.5%), a proxy for low socioeconomic status [57]. A similar number of participants completed the Vioscreen (*n* = 24) and DHQII (*n* = 23) FFQs. Based on the 27 food parameters common between both the FFQs (60% of the total DII parameters), the DII scores were spread across low and higher inflammatory scores with the lowest tertile (Tertile 1) mean of −2.3 ± 0.9 and highest (Tertile 3) mean DII of 3.3 ± 0.5 (Table 1). Socio-demographic characteristics were similar across all three groups (Table 1, *p* > 0.05). A less inflammatory diet was associated with higher intakes of vitamin B12, B6, A, riboflavin, niacin, iron, folic acid, magnesium, and zinc (Table 2, *p* < 0.05). These DII parameters were positively associated with each other (Figure 1, *p* < 0.05). Of the nutrients used to calculate the DII score, the biggest contributors were those vitamins and minerals negatively associated with the DII (Figure 1, *p* < 0.05).

### 3.2. Gut Microbiota Composition and Predicted Metabolic Potential Were Associated with Proinflammatory Diets in Early Pregnancy

**Figure 2:**
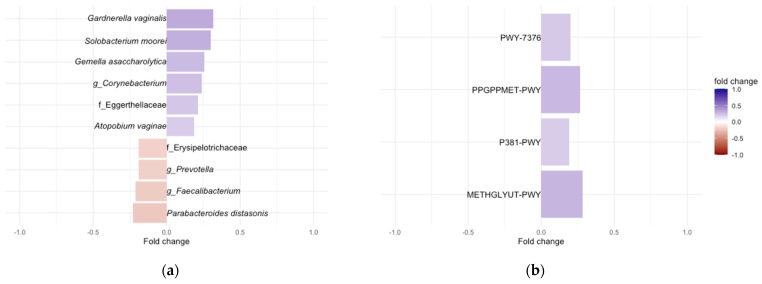
Differentially abundant gut taxa and predicted gut-produced enzymes as a function of DII scores. Top 10 CSS normalized taxa (**a**) and all predicted pathways (**b**) that were identified as statistically significant differentially abundant based on the DII after correction for participant age, estimated gestational weeks (EGA), BMI, and food frequency questionnaire type (DHQII or VioScreen), and sample type (adjusted *p* < 0.05 & *p*-value < 0.01). Taxa names are the lowest identifiable rank. A full list of enriched ASVs can be found in Table S1. *PWY-7376*: Cob(II)yrinate a,c-diamide biosynthesis II; *PPGPPMET-PWYI*: ppGpp metabolism; *P381-PWY*: adenosylcobalamin biosynthesis II (aerobic); *METHGLYUT-PWY*: methylglyoxal detoxification super pathway.

There were no statistically significant differences in the alpha or beta diversities by DII tertile (Figure S2A–D, *p*-value > 0.05). A total of 13 ASVs were identified as differentially abundant in terms of the DII score (Table S1, false discovery rate (fdr)-adjusted *p*-value < 0.05). Among the top 10 ASVs, those mapped to *Solobacterium moorei*, *Gemella asaccharolytica*, *Gardnerella vaginalis*, *Atopobium vaginae*, and unclassified members of the Eggerthellaceae family and the *Corynebacterium* genera were positively associated with the DII (Figure 2, adjusted *p* < 0.05), while those mapped to *Parabacteroides distasonis*, unclassified members of the genus *Faecalibacterium*, *Prevotella*, and the family Erysipelotrichaceae (Figure 2, adjusted *p* < 0.05) were negatively associated with dietary inflammatory potential.

**Figure 3:**
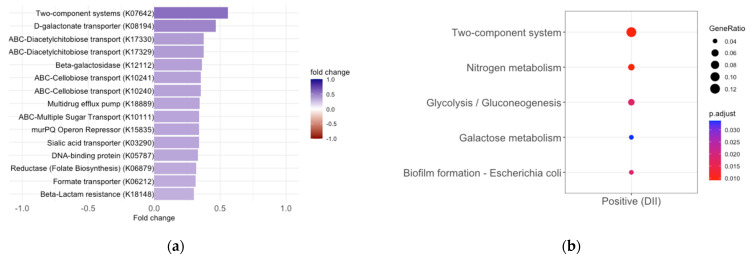
Predicted microbial gene set enrichment analysis in terms of DII scores. (**a**): Top 15 predicted enzymes that were identified as differentially abundant based on the DII (adjusted *p* < 0.05). (**b**): Gene set enrichment of enzymes grouped by those positively (*n* = 192, *p* < 0.05) associated with the DII score. A full list of enriched enzymes can be found in Table S2. A full list of enzymes by gene set term can be found in Table S3.

Next, we examined which PICRUSt2-predicted microbial enzymes and metabolic pathways were associated with the DII scores. We identified four pathways, including two aerobic adenosylcobalamin (vitamin B12) synthesis pathways, a methylglyoxal detoxification pathway, and a nucleotide synthesis pathway (Figure 2b, *p* < 0.01), and 58 enzymes were significantly associated with the DII score (Table S2, fdr-adjusted *p*-value < 0.05). The significantly enriched predicted enzymes were all positively associated with the DII (Table S2), with several being involved in a bacterial two-component system related to multidrug efflux pumps (*K07642*, BaeS) and drug efflux pumps/resistance (*K18889*, *K18148*), nutrient transport (*K17330*, *K17329)*, and galactose degradation and transport (*K10111*, *K12112*, *K0894*) (Figure 3a, fdr-adjusted *p* < 0.05). The gene set enrichment analysis of the DII-associated predicted enzymes before multiple comparisons (*n* = 192, *p*-value < 0.05) also revealed increases in two-component system terms [58] primarily related to nitrogen and sugar metabolism, genes involved in nitrogen metabolism (specifically nitrate reduction to ammonia), biofilm formation, glycolysis, and galactose metabolism (Figure 3b, adjusted *p* < 0.05, Table S3).

### 3.3. Several Individual DII Components Were Associated with Predicted Microbial Enzymes 

**Figure 4:**
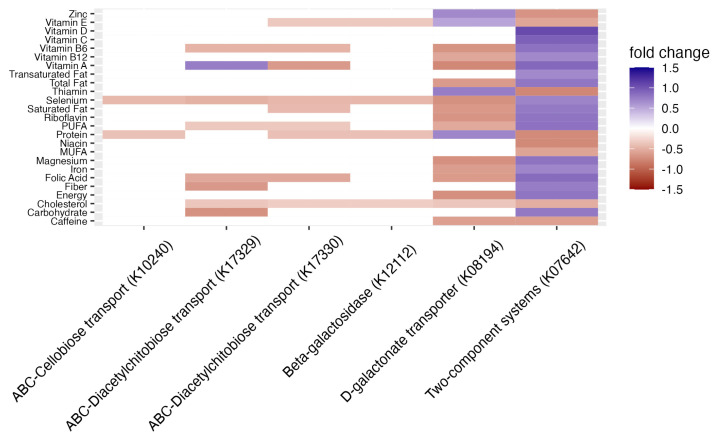
Relationship between predicted enriched enzymes and dietary components. Differential abundance of top 15 predicted enzymes and the 27 dietary components of the DII (adjusted *p* < 0.05). Only significant associations are represented.

Finally, we investigated the relationships between DII components and DII-associated enzymes (Figure 4). Several DII-associated enzymes, such as nutrient transporters, and enzymes pertaining to the galactose metabolism, were also associated with 25 individual DII food parameters including vitamins B12, A, D, and E and cholesterol. *K07642* (a two-component signaling system for efflux pumps) was strongly associated with the largest number of DII components, including a positive relationship with nutrients such as vitamins A, C, and D and negative relationship with others like vitamin E and zinc. The second group of enzymes associated with individual DII nutrient parameters included ABC transporters (*K10240, K17329, K17330*) and galactose metabolism (*K12112*) and metabolite transport (*K01894*) enzymes. These were mostly negatively associated with key perinatal nutrients such as selenium, vitamin B6, and folic acid, some of which were decreased in higher DII individuals (Table 2). 

## 4. Discussion

Dietary intake is an essential aspect of maternal health. Food choice is often related to the dietary preferences of an individual, their environment, and their socioeconomic status. The under- or oversupply of certain nutrients can have direct impacts on maternal health and the growing fetus [59,60]. This study demonstrated that diet inflammatory potential, an indicator of poor diet quality, is associated with lower vitamin and mineral intake, altered maternal gut microbiota composition, and dysregulated microbial metabolic potential in early pregnancy. As diet is one of the main regulators of the gut microbiota [11,12], poor diet quality during pregnancy could disrupt the normal dynamic adaptations of the maternal gut microbiota through altered substrate availability. 

In our study, the overall gut microbiota diversity did not differ in individuals consuming higher inflammatory diets. All DII scores were within the normal limits specified by the DII score authors [29]. While distinct patterns of beta diversity composition in pregnant individuals with better diet quality have been previously reported [61,62], recent microbiome–pregnancy cohorts have not identified alterations in beta diversity based on diet quality [63,64], supporting our study observations. At the taxonomic level, several ASVs varied with dietary inflammatory potential. Higher DII scores were associated with the enrichment of pro-inflammatory bacterial species, including *S. moorei*, a producer of pro-inflammatory sulfur compounds [65], and those associated with inflammatory perinatal conditions such as preterm birth and GDM, including *G. vaginalis*, *A. vaginae* [66] and members of the *Corynebacterium* genera [67]. In contrast, microbiome members that were depleted in individuals reporting high DII scores included known producers of anti-inflammatory SCFAs such as *Faecalibacterium* [68]. This suggests that pro-inflammatory diets are associated with deleterious alterations in the gut microbiota composition. 

The influence of maternal diet quality on the gut microbiota extends to their metabolic potential, as our study reveals a link between the predicted metabolic capabilities of gut microbes in individuals with higher inflammatory diets and community-wide metabolic dysregulation. The Cob(II)yrinate a,c-diamide and adenosylcobalamin biosynthesis metabolic pathway (part of the adenosylcobalamin/vitamin B12 pathway) [69] were increased in participants reporting higher DII scores. Vitamin B12 deficiency can lead to the upregulation of the cytokine TNF-α [70] and has been linked to multiple perinatal disorders including pre-eclampsia and neonate growth retardation [71]. The increase in this bacterial pathway may be related to the insufficient vitamin B12 intake of the high DII group and a subsequent shift toward microbial communities capable of producing this essential vitamin to compensate for the unbalance. The second pathway associated with high DII scores was a microbially regulated methylglyoxal detoxification pathway. Methylglyoxal is a toxic oxidizing substance derived from sugar metabolism, a DII enriched process in this study, and is known to be elevated in perinatal metabolic disorders such as gestational diabetes mellitus [72,73]. Methylglyoxal detoxification can occur through the glyoxalase system [74,75], a common microbial detoxification pathway [76,77]. This finding highlights the pro-inflammatory nature of poor diet quality as well as the compensatory shift in the gut microbiota to reduce toxic metabolic species. Finally, the nucleotides ppGpp (guanosine 3′-diphosphate 5’-diphosphate) and pppGpp (guanosine 3′-diphosphate 5′-triphosphate) are metabolized in the ppGpp metabolism pathway [78]. These nucleotides are known microbial responses to conditions such as nutrient starvation and are associated with virulence mechanisms [78]. 

DII scores were also associated with the upregulation of microbial virulence enzymes, such as drug resistance, biofilm formation, and nitrogen and sugar/galactose metabolism. The sugar and galactose metabolisms, overall, were enriched in individuals reporting high DII scores. Galactose metabolism has been shown to be enriched in perinatal inflammatory conditions such as gestational diabetes [79,80] and specifically associated with elevated methylglyoxal [81]. Notably, *S. moorei* and *G. vaginalis* were both positively linked with DII scores and have been reported to contribute to galactose fermentation [65,82]. The enrichment of microbial multidrug resistant efflux pump enzymes (*K07642,18889, K18148*) could be promoted by host pro-inflammatory diets. Recent work has shown that bacterial multidrug efflux pumps are involved in nutrient signal processing, cellular adaptations to anaerobic respiration, and the colonization of eukaryotic cells [83]. Poor maternal diet quality may promote the expression of these gut microbial enzymes in response to nutrient alterations. The predicted gut microbial enzymes related to galactose metabolism as well as disaccharide transporters (*K10240, K17329, K17330*) were also mostly negatively correlated with vitamins and minerals (i.e., vitamins B12 and A and iron, magnesium, niacin, and zinc) that were decreased in high DII individuals. Taken together with the upregulated microbial pathways, our results suggest that a vitamin- and mineral-depleted perinatal diet is associated with a shift in the gut microbiota toward a more pathogenic/pro-inflammatory community. This work would suggest that vitamin and mineral intake during the gestational period should continue to be of high importance in terms of pregnancy nutrition.

Our cohort primarily comprised low-income Black and Latinx pregnant persons. The intake of highly processed foods is a hallmark of a Western diet, a diet pattern that is more common among disadvantaged minorities in the U.S., as these foods are more affordable and attainable for individuals with high financial burdens [84]. Previous studies on large perinatal cohorts, such as the 30-year longitudinal AVON study, have shown that women with lower access to high quality foods, have decreased vitamin and mineral intake [6]. Our results support the hypothesis that poor diet quality is linked to insufficient vitamin and mineral dietary intake and is accompanied by pro-inflammatory adjustments in the gut microbiome composition and metabolic structure. 

## 5. Strengths and Limitations

Our work focused on an understudied population at high risk of multiple health disorders, such as hypertension and GDM [6,85]. Associations between diet inflammatory potential and gut microbiota during pregnancy are underexplored, and our research indicates that there is a significant link between microbial composition and metabolic functions and dietary inflammatory potential. A notable limitation in using the DII for this work is it utilizes total nutrient values such as total protein. However, the gut microbiota may respond differently to different types of dietary protein (animal vs. plant). Our work could be further improved in several ways: (1) by employing a more comprehensive dietary assessment approach that can assess all the 45 dietary parameters to calculate the DII instead of just a portion of them (27 used for this study); (2) by including a larger sample of a more diverse population in terms of DII scores that is followed longitudinally to determine the effects of the gut microbiome later in pregnancy and perinatal disease development on the DII; (3) by employing a single-stool sampling method; (4) by utilizing the same diet assessment for all participants and at the same collection time; (5) by employing sequencing technologies that enable the measurement of the abundance of microbial genes, such as shotgun sequencing (metagenomics) instead of relaying in metabolic predictions; (6) by further characterizing the host immune and metabolic profiles; and (7) by including dietary data from multi-site centers to reflect different communities diets as ours was primarily limited to Western diets.

## 6. Conclusions

A pro-inflammatory diet, measured using the DII, characterized by the low intake of vitamins B12, B6, and A and iron, magnesium, niacin, riboflavin, and zinc, during early pregnancy is associated with a pro-inflammatory shift in the gut microbiota and metabolism as indicated by increases in the galactose metabolism and methylglyoxal detoxification and multidrug efflux pump expression. The further characterization of gut metabolic status as a function of dietary alterations can provide opportunities for future research and targeted intervention strategies for at-risk perinatal populations.

## Figures and Tables

**Table 1:** Study cohort demographic characteristics did not differ as a function of the DII scores. Participants were stratified into DII tertiles. There were no differences in study characteristics by DII tertile (*p* > 0.05).

		Tertile 1	Tertile 2	Tertile 3	*p*
DII	Mean (SD)	−2.3 (0.9)	1.0 (0.9)	3.3 (0.5)	
Gestational Weeks	Mean (SD)	11.2 (3.7)	11.2 (2.9)	10.0 (2.6)	0.46
Age	Mean (SD)	29.4 (7.5)	28.6 (5.3)	30.2 (5.5)	0.74
BMI	Mean (SD)	29.4 (7.3)	30.4 (6.6)	28.5 (7.7)	0.76
Race/Ethnicity	Hispanic	2 (13.3)	4 (25.0)	1 (6.2)	0.31
	Non-Hispanic Black	9 (60.0)	6 (37.5)	6 (37.5)	
	Other/Unreported	4 (26.7)	6 (37.5)	9 (56.2)	
Health Insurance	Federal Aid	13 (86.7)	10 (62.5)	12 (75.0)	0.3
	Private	2 (13.3)	6 (37.5)	4 (25.0)	
Education	Above College	1 (6.7)	4 (25.0)	5 (31.2)	0.24
	Below College	5 (33.3)	4 (25.0)	7 (43.8)	
	College	9 (60.0)	8 (50.0)	4 (25.0)	
Employment	Employed Part/Full Time	10 (66.7)	8 (50.0)	9 (56.2)	0.64
	Unemployed	5 (33.3)	8 (50.0)	7 (43.8)	
Income	$31–76 k	2 (13.3)	3 (18.8)	2 (12.5)	0.83
	$76 k+	1 (6.7)	3 (18.8)	2 (12.5)	
	<$31 k	12 (80.0)	10 (62.5)	12 (75.0)	
Relationship Status	Married/Relationship	7 (46.7)	11 (68.8)	10 (62.5)	0.44
	Single	8 (53.3)	5 (31.2)	6 (37.5)	
Planned Pregnancy	No	6 (40.0)	1 (6.2)	2 (12.5)	0.13
	Unreported	7 (46.7)	10 (62.5)	11 (68.8)	
	Yes	2 (13.3)	5 (31.2)	3 (18.8)	

**Table 2:** Differences in nutritional intake by DII tertile. Reported mean (SD) nutrient values were normalized using the total energy intake per day (kcal/day). Vitamin A is reported in retinol equivalents (REs).

	Tertile 1	Tertile 2	Tertile 3	*p*
Alcohol	0.1 (0.3)	0.1 (0.2)	0.1 (0.5)	0.82
Vitamin B12 (μg)	3.2 (1.1)	2.9 (1.2)	2.2 (0.9)	0.04
Vitamin B6 (mg)	1.2 (0.4)	1.0 (0.3)	0.9 (0.2)	0.03
β Carotene (μg)	2094.3 (1552.1)	1547.3 (1698.9)	1551.1 (1112.2)	0.5
Caffeine (g)	0.0 (0.0)	0.0 (0.0)	0.0 (0.0)	0.49
Carbohydrate (g)	127.5 (25.9)	129.0 (28.0)	128.2 (34.8)	0.99
Cholesterol (mg)	140.6 (66.9)	145.8 (64.5)	163.6 (117.6)	0.74
Energy (kcal)	3001.5 (954.9)	1801.1 (409.1)	970.5 (344.7)	
Total fat (g)	39.6 (9.9)	40.3 (9.5)	40.1 (11.8)	0.98
Fiber (g)	11.0 (2.5)	10.1 (3.3)	9.1 (2.2)	0.15
Folic acid (μg)	165.3 (87.0)	165.8 (86.0)	134.1 (74.9)	0.47
Iron (mg)	9.2 (3.5)	7.9 (2.8)	6.3 (1.7)	0.02
Magnesium (mg)	173.4 (35.6)	147.1 (35.7)	144.4 (41.7)	0.07
MUFA (g)	0.0 (0.0)	0.1 (0.0)	0.0 (0.0)	0.56
Niacin (mg)	11.8 (3.2)	10.1 (2.1)	9.1 (2.8)	0.03
Protein (g)	39.3 (6.3)	35.6 (7.0)	36.1 (11.7)	0.44
PUFA (g)	8.1 (2.4)	7.9 (2.7)	8.2 (4.9)	0.96
Riboflavin (mg)	1.3 (0.4)	1.1 (0.4)	1.0 (0.3)	0.04
Saturated fat (g)	13.5 (3.6)	14.2 (4.1)	13.4 (4.4)	0.85
Selenium (μg)	52.5 (10.4)	49.5 (11.6)	51.9 (19.8)	0.82
Thiamin (mg)	0.9 (0.3)	0.8 (0.2)	0.8 (0.2)	0.19
Trans fat (g)	1.7 (0.5)	1.8 (0.6)	1.6 (0.7)	0.74
Vitamin A (RE)	537.0 (191.2)	437.0 (191.1)	368.2 (150.0)	0.04
Vitamin C (mg)	87.8 (42.8)	71.7 (47.2)	66.7 (54.0)	0.46
Vitamin D (μg)	4.0 (2.0)	3.6 (2.0)	2.6 (1.7)	0.15
Vitamin E (mg)	5.2 (2.1)	3.9 (1.2)	4.1 (1.9)	0.11
Zinc (mg)	6.6 (1.9)	5.9 (1.4)	5.1 (1.4)	0.04

## Data Availability

Raw 16s amplicon sequencing data will be uploaded to the NCBI SRA database before publication. The raw Vioscreen and DHQII data, de-identified metadata and DIIs, and all R scripts are available online at https://github.com/LabBea/Perinatal_DII, accessed on 19 February 2024.

## Supplementary Materials

The following supporting information can be downloaded at https://www.mdpi.com/article/10.3390/nu16070935/s1: Table S1: DII differentially abundant ASVs using zero-inflated generalized linear models; Table S2: DII differentially abundant microbial enzymes using zero-inflated generalized linear models; Table S3: Microbial enzymes per term identified via gene set enrichment by DII; Figure S1: Participant flowchart; Figure S2: Alpha and beta diversities were not associated with assessment by DII score.
