# Supplementary material for: Inflammatory Dietary Potential Is Associated with Vitamin Depletion and Gut Microbial Dysbiosis in Early Pregnancy
Source: Nutrients. 2024 Mar 24;16(7):935. doi: 10.3390/nu16070935 (PMC11013194; doi:10.3390/nu16070935)
Supplement: Supplementary file 1 [file nutrients-16-00935-s001.zip › Supplemental Figures and Legends.pdf]

Supplementary Materials

Supplemental Table S1: DII differentially abundant ASVs using zero-inflated generalized linear models. Corrected by subject age, gestational weeks, sample type, FFQ type, and BMI (adjusted p-value < 0.05).

Attached excel file

Supplemental Table S2: DII differentially abundant microbial enzymes using zero-inflated generalized linear models. Corrected by subject age, gestational weeks, sample type, FFQ type, and BMI. Microbial enzymes were all increased (N=38) by DII. We employed the KEGG database as a reference.

Attached excel file

Supplemental Table S3: Microbial enzymes per term identified by Gene set enrichment by DII. Microbially enzymes that were positively associated with DII before multiple comparison adjustments (N=194).

Attached excel file

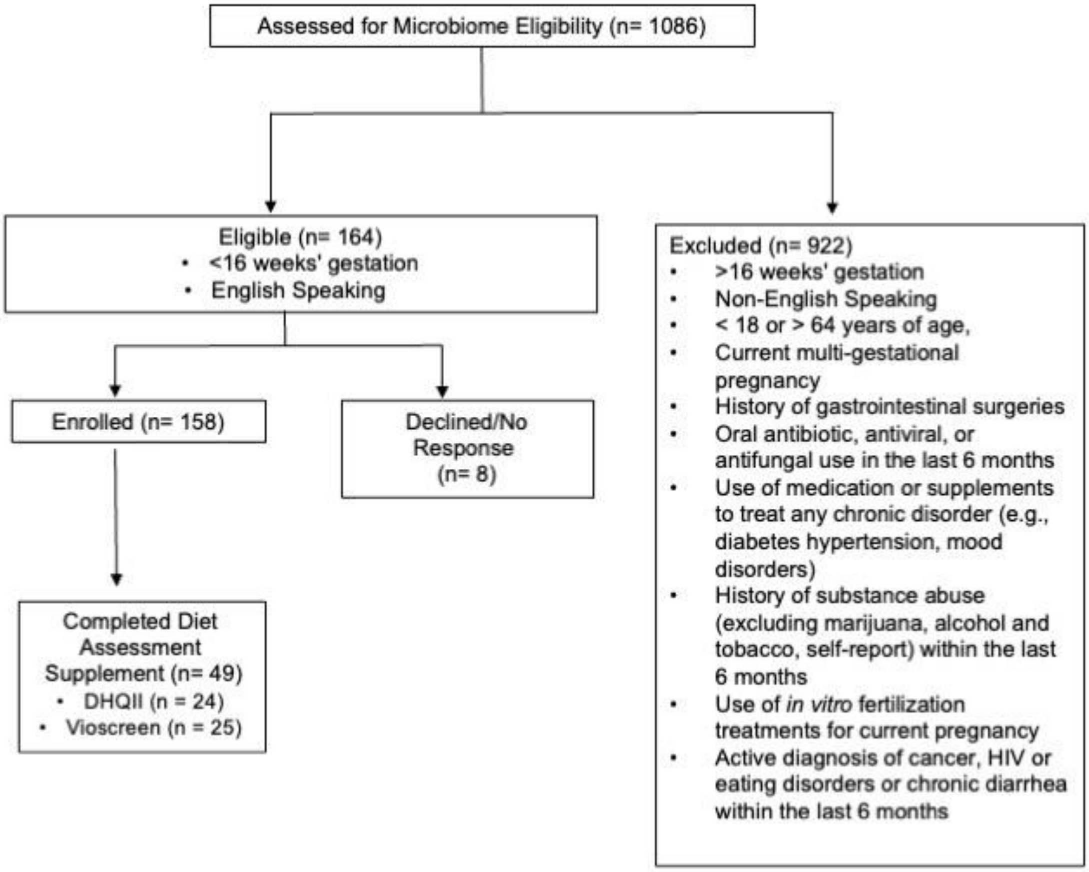

Supplemental Figure S1: Participant Flow Chart. A total of 1086 pregnant persons were screened for potential enrollment in the Microbiome sample collection. 922 were ineligible based on study exclusion criteria. 164 were eligible and 158 enrolled. Of those, 47 completed a one month recall food frequency questionnaire (DHQII or Vioscreen) in early pregnancy.

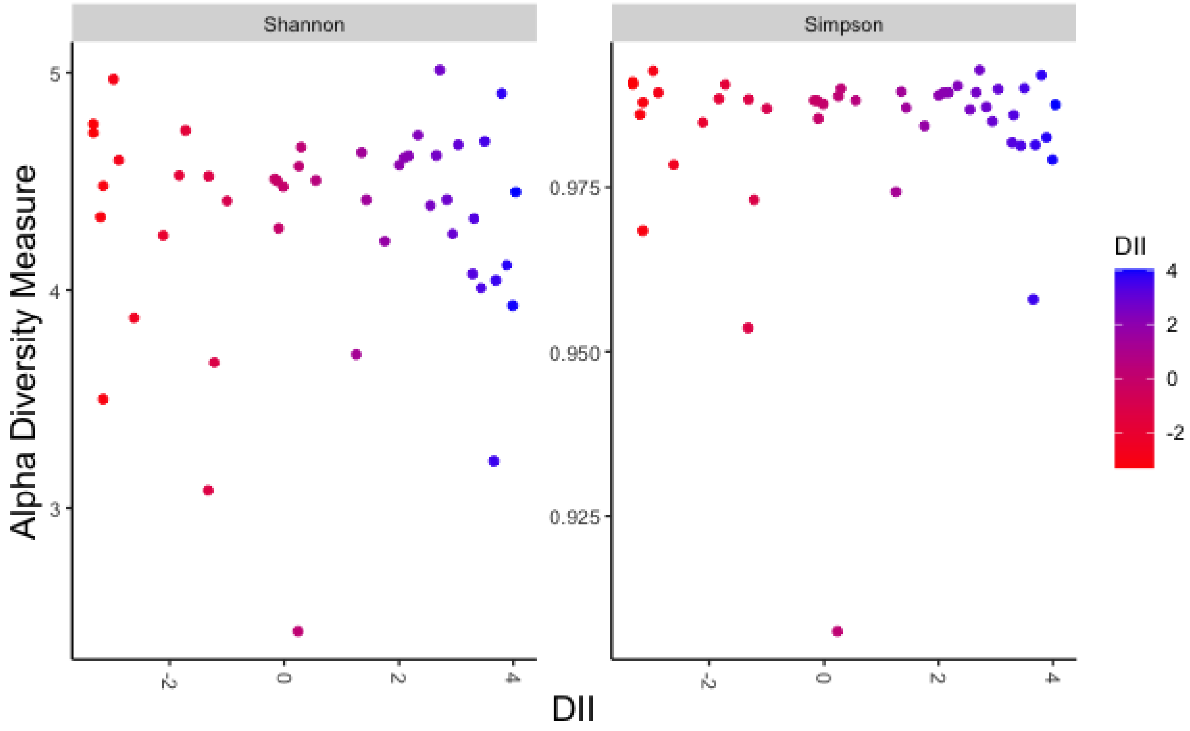

(a)

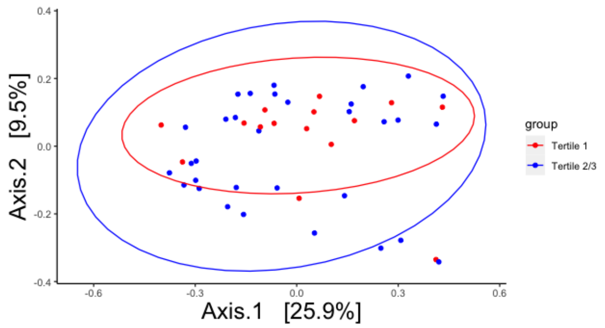

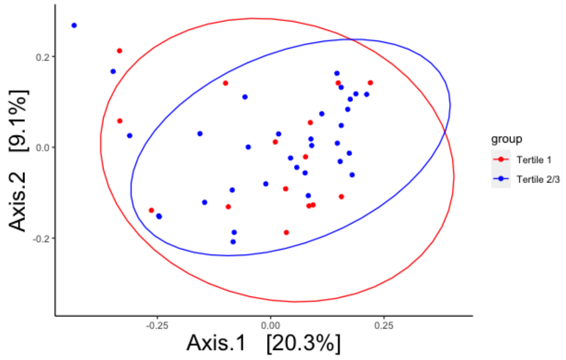

(b)

(c)

Supplemental Figure S2: Alpha and beta diversity were not associated with assessment by DII score. A: Shannon and Simpson indexes as a function of DII scores. B: DII tertile (Wilcox Rank Sum p-value > 0.05). B: Beta diversity measured by Bray Curtis distance as a function of DII tertiles (PERMANOVA, p-value > 0.05). C: Beta diversity measured by UniFrac distance. Ellipses represent Tertile 1 and Tertile 2/3.
